# Study of the geometry of open channels in a layer-bed-type microfluidic immobilized enzyme reactor

**DOI:** 10.1007/s00216-021-03588-x

**Published:** 2021-08-10

**Authors:** Cynthia Nagy, Robert Huszank, Attila Gaspar

**Affiliations:** 1 Department of Inorganic and Analytical Chemistry, University of Debrecen, Egyetem ter 1, Debrecen, 4032 Hungary; 2 Institute for Nuclear Research (Atomki), P.O. Box 51, Debrecen, 4001 Hungary

**Keywords:** PDMS, Enzyme reactor, Peptide mapping, COMSOL, Layer bed, Saliva

## Abstract

This paper aims at studying open channel geometries in a layer-bed-type immobilized enzyme reactor with computer-aided simulations. The main properties of these reactors are their simple channel pattern, simple immobilization procedure, regenerability, and disposability; all these features make these devices one of the simplest yet efficient enzymatic microreactors. The high surface-to-volume ratio of the reactor was achieved using narrow (25–75 μm wide) channels. The simulation demonstrated that curves support the mixing of solutions in the channel even in strong laminar flow conditions; thus, it is worth including several curves in the channel system. In the three different designs of microreactor proposed, the lengths of the channels were identical, but in two reactors, the liquid flow was split to 8 or 32 parallel streams at the inlet of the reactor. Despite their overall higher volumetric flow rate, the split-flow structures are advantageous due to the increased contact time. Saliva samples were used to test the efficiencies of the digestions in the microreactors.

**Graphical abstract:**

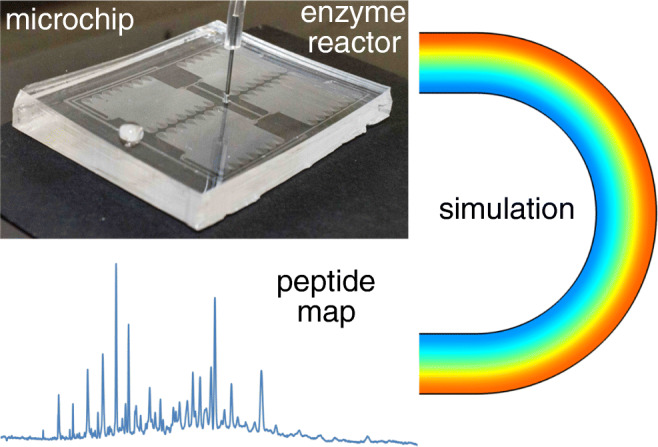

**Supplementary Information:**

The online version contains supplementary material available at 10.1007/s00216-021-03588-x.

## Introduction

The aim of proteomic research is to provide a comprehensive characterization of the proteome. Although the complete protein profiling of living organisms still faces analytical challenges, the emergence of shotgun proteomics greatly enhanced the success of such endeavours [1]. In a typical “shotgun” workflow, the sample is mixed with a proteolytic enzyme (e.g. trypsin) to cleave the proteins into smaller peptides; the resulting peptides are then separated by high-performance liquid chromatography (HPLC) [2] or capillary electrophoresis (CE) [3] and finally analysed by tandem mass spectrometry (MS/MS). The standard, in-solution digestion protocol suffers from long incubation times (2–16 h) as the enzyme can only be used in high dilution (protein:trypsin = 20–100:1) to minimize autolysis [4]. Extended digestion times can be considered the bottleneck for fast, high-throughput MS-based analysis. Trypsin autolysis, however, can be suppressed effectively by binding the enzyme to solid supports, offering accelerated digestions (~ few minutes) [5]. To this end, the development and application of microfluidic immobilized enzyme reactors (μ-IMERs) have attracted much attention. Enzymes are most commonly attached to supports via adsorption [6], covalent bond [7], and bioaffinity linkage [8]. An important feature of such microfluidic devices (MD) is their high surface area-to-volume ratio (*S*/*V*), which can boost digestion efficiency by increasing the occurrence of enzyme–substrate interactions.

Despite the inherently high specific surface area of MDs, considerable efforts have been made to further increase the *S*/*V* ratio by integrating porous membranes [6, 9], particles [10, 11], monoliths [12–14], and magnetic microspheres [15, 16] into the channels. Each strategy has their benefits and drawbacks. Accommodation of particles in a microfluidic channel is in the interest of chromatography experts, as well. However, particle retention is an exhaustive task both in conventional and microfluidic platforms; furthermore, packing heterogeneity cannot be prevented, leading to peak broadening. An elegant way to circumvent this issue is to form collocated monolith support structures (COMOSS) [17] or pillars [18] by microfabrication techniques adapted from the microelectronic industry. In this way, excellent homogeneity was achieved while maintaining a sufficiently high *S*/*V* ratio. Although the authors used this pillar array platform for LC separations, the underlying motivation is valid for the development of μ-IMERs, as well.

The present study focuses on wall-coated (or layer-bed type) μ-IMERs and the possibilities of advancing catalytic performance in such systems. Wall-coated μ-IMERs are the simplest and probably possess the lowest enzyme loading capacity in comparison with packed or monolithic μ-IMERs, as in this case only the inner wall of the microchannel serves as solid support for immobilization. More recently, the utilization of multi-channel capillaries has attracted attention [19, 20] as possible candidates for enzyme housing. Such a capillary embodies an array of microchannels, surmounting the shortcomings of single-channel wall-coated IMERs in terms of *S*/*V* ratio. In order to increase the available surface area, the pillar array structure mentioned above can be utilized [21, 22] as well as the modification of the inner wall (e.g. sol–gel matrix [23], cross-linked enzyme membrane [24]). The long diffusion length can also be minimized by decreasing the channel diameter, as realized by Foret et al., who have developed an excellent enzymatic reactor inside a 10-μm-inner-diameter (i.d.) capillary [7]. While most immobilization techniques described in the literature require a multi-step manipulation of the channel surface, our group has recently proposed the method of spontaneous trypsin adsorption directly on the channel surface of a polydimethylsiloxane (PDMS) microchip, providing one of the simplest μ-IMER setups, where trypsin is adsorbed directly on the unmodified PDMS channel walls via hydrophobic interactions [25]. Previously, the quasi-irreversible adsorption of proteins onto PDMS had been confirmed utilizing surface plasmon resonance spectroscopy [26]. A brief evaluation of trypsin adsorption can be found in the supplementary material as Fig. ESM-1. PDMS is often at the centre of dispute as to whether it has any real advantage over other sturdier materials, but the very problems usually associated with PDMS (adsorptivity, hydrophobicity, porosity) are actually exploited in the IMER presented. The digestion with this microreactor using a layer-bed-type immobilized enzyme reactor (empty channels) requires less than 10 min, while in-solution digestion takes 16 h [25, 27].

The goal of the current work was to study such open channel geometries in a layer-bed-type immobilized enzyme reactor system with computer-aided simulations. The curves of the channel may either have a considerable or a negligible effect, mainly depending on the channel diameters and the flow rate. Since in microfluidic enzyme reactors applied for proteomic studies the required volumetric flow rate and the channel width/height are in a relatively narrow range, the effect of the curves was examined for our particular IMER design. The simulations allow us a better understanding of laminar liquid flow in empty channel geometries, granting us the possibility to fully harness the proteolytic potential of these systems. To put the designed microchannels to a test, protein samples bearing clinical significance and challenging complexity were used for digestions.

## Materials and methods

### Materials

Analytical-grade reagents were used. Urea, dithiothreitol (DTT), iodoacetamide (IAM), NH_4_HCO_3_, and formic acid (FA) stock solutions (all Sigma products, St. Louis, MO, USA) were prepared in double-deionized water (Elix-3, Millipore, Darmstadt, Germany). Porcine pancreas trypsin (Type IX-S, lyophilized powder, Sigma) solutions were freshly prepared before each experiment. Human saliva was digested to examine the efficiency of the μ-IMERs. Phosphate buffer (PB) electrolyte, isopropanol, methanol, and acetonitrile were purchased from VWR (Radnor, PA, USA).

For microchip fabrication, SU-82025 photoresist and SU-8 developer solution (1-methoxy-2 propyl) acetate) were acquired from Microchem (Newton, MA, USA). The PDMS silicone elastomer kit (Sylgard 184) was purchased from Dow Corning (Midland, MI, USA).

### Flow simulation

COMSOL Multiphysics (Burlington, MA, USA) software was utilized to simulate liquid flow behaviour in the microchannels. This is a finite element (FEM) analysis-based simulation software. Software version 5.3a was used with laminar flow and transport of diluted species modules to simulate liquid flow behaviour. The flow velocity was fixed at 3 × 10^−3^ m/s, the diffusion coefficient was set to 6.1 × 10^−11^ m^2^/s, and the concentration of one of the inlets was set to 4.3 × 10^−5^ mol/L, in all cases. The mesh size was set to extremely fine.

### Preparation of the PDMS microreactor

The microfluidic chips were fabricated by means of soft photolithography [28]. The channel patterns designed with AutoCAD software were printed with a high-resolution printer (Keppont Ltd., Debrecen, Hungary). A 3″ silicon wafer was coated with negative-type photoresist (SU-8) using a spincoater (3000 rpm, 30 s). Following soft bake (95 °C, 15 min), the photoresist-coated wafer was exposed to UV light (365 nm, 10 min) through the printed lithographic mask. After post-exposure bake (95 °C, 5 min), the unexposed areas were dissolved by rinsing with SU-8 developer solution. The attained SU-8 pattern on the wafer served as the mould, from which inverse replicas were made using PDMS. A mixture containing PDMS oligomer and curing agent in a 10:1 ratio was poured onto the mould. The mould was then placed into a vacuum chamber to eliminate air bubbles. After curing (65 °C, 60 min), the PDMS was stripped off the mould and cut to size, and holes (300 μm diameter) were pierced at the ends of the channels (for liquid connections) and finally irreversibly sealed onto another PDMS slab following oxygen plasma treatment (PDC-32G, Harrick, Ithaca, NY, USA).

A peristaltic pump (IPC, Ismatec, Cole-Palmer, IL, USA) was used to transfer the reagents and protein samples through the microreactors. The peristaltic pump’s tubing (ID, 0.19 mm; Tygon, Cole-Palmer, IL, USA) was connected to the inlet port of the channel.

### Enzymatic digestion of saliva samples

Human saliva samples were obtained from a healthy female volunteer. Sampling was carried out according to the spitting method [29] after having abstained from food and beverage consumption for at least 2 h. Whole saliva collected into a 2-mL-volume Eppendorf tube was centrifuged (2700 × g, 20 min). Supernatant was freeze-dried. The pretreatment of saliva samples for the tryptic digestion was executed as follows: ~ 1 mg lyophilisate was dissolved in 6.6 μL 25 mM NH_4_HCO_3_. Twenty microlitres of 8 M urea solution (30 min, room temperature) was added to unravel the tertiary structure of the proteins. For the reduction of the disulfide bonds, 2.66 μL 100 mM DTT was used (1 h, 37 °C). Adding 2.66 μL 200 mM IAM (alkylating agent) to the solution (45 min, room temperature in the dark), the recombination of disulfide bonds was precluded. Finally, 133 μL 25 mM NH_4_HCO_3_ was added to the mixture. The prepared samples were stored at − 20 °C until digestion. Digestions were performed the conventional way (in-solution) and via μ-IMER.

#### In-solution digestion

6.6 μL freshly prepared 1 mg/mL trypsin solution was pipetted into the saliva samples. Reaction was stopped after overnight incubation (16 h, 37 °C) by the addition of 1% FA to a 0.1% FA final concentration.

#### Digestion in μ-IMER [25]

For immobilization, freshly prepared 20 mg/mL trypsin solution was flushed through the PDMS channels (2 μL/min, 10 min). Unbound trypsin was removed from the channel by flushing with 25 mM NH_4_HCO_3_ (2 μL/min, 10 min). For the digestion, 10-μL aliquots of the sample were transported through the microreactor at a flow rate of 0.65 μL/min (contact time, ~ 2 min; room temperature). The peptide mixture at the outlet was collected for the subsequent CE-UV and CE-MS measurements. 1.5 μL 1% FA was added to the effluent sample to inhibit accidental tryptic activity (in case of trypsin leaching).

### Capillary electrophoresis

The separation of peptides was performed with a 7100 model CE instrument (Agilent, Waldbronn, Germany) using on-capillary UV and MS (maXis II UHR ESI-QTOF MS instrument, Bruker, Bremen, Germany) detection. Hyphenation was achieved with a CE-ESI Sprayer interface (G1607B, Agilent). Sheath liquid was transferred with a 1260 Infinity II isocratic pump (Agilent). CE instrument was operated by OpenLAB CDS Chemstation (Agilent) software and the MS instrument was controlled by otofControl version 4.1 (build: 3.5, Bruker).

Fused silica capillaries of 90 cm × 50 μm i.d. (Polymicro, Phoenix, AZ, USA) were used for the separations. Sample solutions were introduced at the anodic end of the capillary; the applied voltage was 25 kV. The capillaries were preconditioned with the background electrolyte (BGE) for 5 min and postconditioned with methanol, acetonitrile, and BGE for 2 min each. In the case of CE-UV determinations, the BGE used was phosphate buffer (PB) (100 mM, pH = 2.2); hydrodynamic sample introduction (50 mbar, 10 s) was used for sample injection, and peptides were detected at *λ* = 200 nm. For CE-MS measurements, the BGE consisted of formic acid (FA) (1 M, pH = 1.9), and larger sample loading was performed (50 mbar, 120 s). The sheath liquid was isopropanol:water = 1:1 with 0.1% FA and applied with 6 μL/min flow rate. MS was operated in positive ionization mode; 0.5 bar nebuliser pressure, 180 °C dry gas temperature, 4 L/min dry gas flow rate, 4500 V capillary voltage, 500 V end plate offset, 3 Hz spectra rate, and 50–2200 *m*/*z* mass range were applied. Collision-induced dissociation (CID) was used to produce fragment ions. The MS/MS spectra rate was 1–4 Hz. Na-formate calibrant injected after each run enabled internal *m*/*z* calibration. Electropherograms were processed by OpenLAB CDS Chemstation (Agilent) software; mass spectra were processed by Compass DataAnalysis version 4.4 (build: 200.55.2969, Bruker). Protein identification was carried out with Byonic software (Protein Metrics, Cupertino, CA, USA). Digestion specificity was set to fully specific allowing up to 1 missed cleavage; precursor mass tolerance was 10 ppm. Carbamidomethylation at Cys as fixed modification, deamidation at Asn and Gln, and formylation at N-term were set as variable modifications.

## Results and discussion

### Increasing the surface-to-volume ratio

For enhancing the efficiency of an enzyme reactor, the main parameters to be tuned are the total surface and the surface-to-volume ratio (*S*/*V*) of the channel system as well as the residence time of the analyte in the close vicinity of the wall covered by the enzyme; i.e. these parameters are to be maximized. There are several ways to largely increase the total surface and the *S*/*V* of a channel system. Recently, the arrangement of pillars [22] or integration of a highly porous medium [6–9, 12–14] in a channel has seemed to be very efficient and is preferred. However, the simplest channel pattern is obviously an empty channel, where the total surface and the *S*/*V* can easily be adjusted by changing the dimension (mainly the width and the length) of the channel. In a recent study, we have shown that *S*/*V* values and total surface similar to those of channels with pillars can be achieved in empty channels as well [22]. The pillars in a microfluidic system do not cause turbulence or diversion of the liquid stream, which could allow components to travel close to the wall covered by the immobilized enzyme (Fig. ESM-2).

**Fig. 1:**
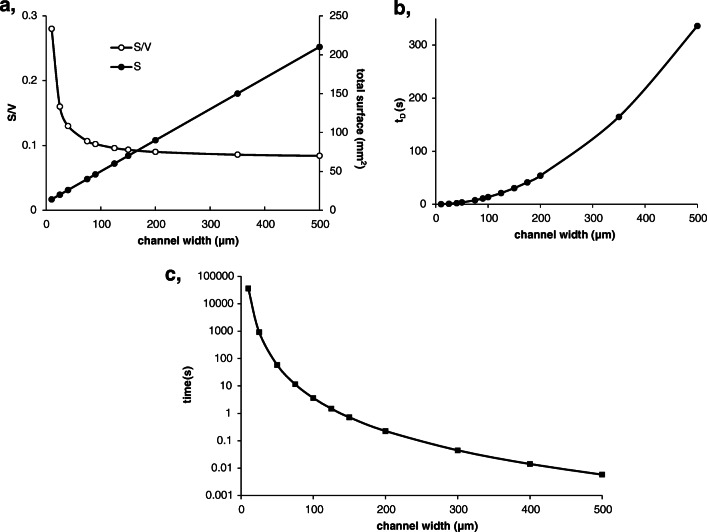
The dependence of surface-to-volume ratio (*S*/*V*) and total surface (*S*) on the channel width (**a**), diffusion time (*t*_D_) as a function of channel width (*D*_albumin_ = 6.1 · 10^−11^ m^2^/s) (**b**), and the time it takes to transport 10 μL sample solution through the channels of different widths (*p*, 2 bar; *L*, 20 cm; liquid, water) (**c**)

While the *S*/*V* values largely increase with the narrowing of the empty channel, the total surface linearly increases with the width of the channel (Fig. 1a). The use of too wide (> 500 μm) channels is outside the scale of microfluidics, but the channels narrower than 10 μm are difficult to fabricate in an average microfluidic lab. (Whereas Quake stated that even patterns of tens of nanometre range can be formed into polydimethylsiloxane using a proper template [30], the works published subsequently reported channels with 20–500 μm width using photolithography.) On the other hand, the required pumping pressure is reversely proportional with the 4th power of the channel width.

Wider channels are less advantageous for μ-IMERs because the analyte molecules are more restricted in reaching the surface immobilized with the enzymes. In μ-IMERs, the flow is strongly laminar, so the molecules can approach the wall only with diffusion. Proteins (as the analytes for μ-IMERs) are large molecules; therefore, their diffusion rate is very small (e.g. *D* = 6.1^.^10^−11^ m^2^/s for albumin). While in a 100-μm-wide channel it takes 20 s for a molecule of albumin to travel from the middle to the surface, only 1.3 s is required in a 25-μm-wide channel (Fig. 1b). That means if the albumin sample is pumped through the μ-IMER faster than 20 s, a small portion of the protein has no chance for enzyme digestion.

However, the application of too narrow channels would limit the volume of the sample to be digested. Ten microlitres volume of sample is minimally needed for an MS measurement even if it is hyphenated with HPLC or CE. Figure 1c shows the time needed to transport 10 μL sample solution through the channels of different widths. Using 10 μm channel width, the sample should be transported for more than 10 h, while only 15 min is needed in a 25-μm-wide channel and only a few seconds in a 100-μm-wide channel (*p*, 2 bar; *L*, 20 cm). Based on the considerations above, there is no reason to decrease the channel width below 25 μm or increase it over 100 μm; therefore, the recommended range of the channel width is 25–75 μm.

Since the essential requirement for the efficient operation of a μ-IMER is that components should reach the wall, the diffusion/radial motion of the transported components in the channel was studied by COMSOL simulations. We studied a straight channel where water and 43 μM albumin were introduced into the inlet as two parallel streams at 1:1 ratio (“each above each”) and pumped these liquid streams with the same speed (3 mm/s). COMSOL simulations (Fig. ESM-3 and Fig. ESM-4) show the concentration distribution of a protein across channels of varying widths (10, 25, 50, 100 μm). Four cross-sectional channel segments of 50 μm width at 0.005, 1, 9.95, and 19.95 mm distance were magnified for better visibility. The simulations demonstrate that while the concentration distribution of the component is completely homogeneous in the channel of 10 μm width (all components can contact the wall) after 1 mm length, in the channel of 100 μm width the distribution is not homogeneous even after 20 mm length (a considerable part of the components cannot reach the wall). In the case of the 25- and 50-μm-wide channels, the mixing of the solutions becomes completed within a 20-mm distance.

Mixing in the channel is defined by the flow rate, as well. Concentration distributions corresponding to three different linear flow velocities (0.3–3–30 mm/s) in a 25-μm-wide channel differ considerably (Fig. ESM-5 and Fig. ESM-6). Although the linear speeds examined (commonly used in microfluidics) are largely different, the flow is still strongly laminar (Re, 0.0084–0.84) in each case. When the speed is only 0.3 mm/s, the concentration distribution becomes homogeneous after a length of 1 mm. Not surprisingly, similar homogeneous distribution can be obtained after 10 mm with 3 mm/s flow rate because the residence times of the component in the channel are identical. Theoretically, in straight channels, no difference can be expected in the digestion efficiency between long channel–high flow rate and short channel–low flow rate arrangements. However, the generation of a very low flow rate in a stable, constant way can count as a technical difficulty (e.g. 0.3 mm/s equals 0.009 μL/min in a 25-μm-wide and deep channel, which is a lower rate than the lowest available flow rate with a typical peristaltic pump (~ 0.2 μL/min).

### Effect of curves

Fluid flow in microfluidic channels is known for its laminar behaviour, where streamlines do not cross paths. Special channel configurations, however, induce passive mixing [31–42]. In such cases, the geometric features or obstructions in the channel are the source of the mixing phenomenon. It has been reported that curves in a microchannel generate a spiralling fluid flow, the magnitude of which can be characterized by the Dean number [31–35]. The mixing effect of such secondary flow is pronounced in systems where Re > 10 [34]. Raising the Re (hence, the flow rate) increases the value of the Dean number, which is favourable if a homogenous concentration distribution is desired. Some studies show a considerable effect of the curves on the disturbance of liquid flow [34–36]. However, other papers claim that the curves have only a minor effect on the dynamics/flow profile of the liquid [34, 37], therefore, different types (passive or active) of mixing/diverting are still required for efficient mixing [38, 39]. In addition to the Dean vortices, expansion vortices can also form in channels where there is an abrupt increase in cross-sectional area at the curvature, creating a multivortex field [43]. Therefore, the impact curvatures have on mixing is highly variable. Depending primarily on the channel diameter and the flow rate, curves may have either an appreciable or a negligible effect. In microfluidic enzyme reactors applied for proteomic studies, the required volumetric flow rate and the channel width/height can be in a relatively narrow range, because (i) at least 10–50 μL sample should be gained at the reactor outlet, (ii) the proteins present in the solution should reach (diffuse to) the wall of the reactor, and (iii) the digestion (residence time of the sample in the channel) should take less than a few minutes. Therefore, it is not obvious that the application of curves could lead to advantages for digestion in a microfluidic IMER. This is why we intended to study the effects of the curves.

In the case of microfluidic IMERs, two—counteractive—key objectives have to be fulfilled: (i) the exploitation of the radial motion of components toward the channel wall (i.e. an intrinsic passive mixing due to curvilinear channel geometry) and (ii) low flow rate for longer residence time (for increased contact time between enzyme and substrate). In view of these aims, finding a delicate balance between these two contributing factors was a priority. Our systems can be described with Re < 1 (in the case of 0.65 μL/min, the Reynolds numbers are 0.62 and 0.21 for the 25-μm- and the 75-μm-wide channels, respectively), thus only slightly facilitating the development of Dean vortices. Since the Re can be higher if the flow rate is increased, in order to provide sufficient time for enzyme–substrate interaction, the channel length should be increased. Based on these theoretical considerations, with the aid of COMSOL simulations, we studied the extent of the impact curves in the microfluidic IMERs have on the mixing effect and thus the digestions.

Firstly, as a simple case, a straight channel (20 mm long, 100 μm wide) with a single 180° bend was used for studying the effect of one curvature on mixing/diffusion. The concentration distributions at the different positions (fore part, middle, and rear part) of the curve and close to the inlet and outlet ends of the channel were compared (Fig. ESM-7). Although no visually obvious differences in the distributions can be seen in the curvature section, the concentration gradient diagrams demonstrate a little change of the concentration differences in the cross section of the channel in the curvature compared to that obtained for the straight channel before the curve. The concentration differences along the cross section of the channel before and after the curve (0.78 mm length) were 0.026 mM and 0.022 mM, respectively. By contrast, regarding the 9.55-mm-long straight part of the channel, the concentration differences were 0.043 mM and 0.026 mM, respectively. Comparing the change in concentration distributions for each case, it can be concluded that the curvature induces a mixing effect almost three times larger than a straight channel per unit length. Similar conclusions can be reached if the changes of concentration gradients are compared in the case of a straight channel and a channel including a curve (Fig. ESM-8).

**Fig. 2:**
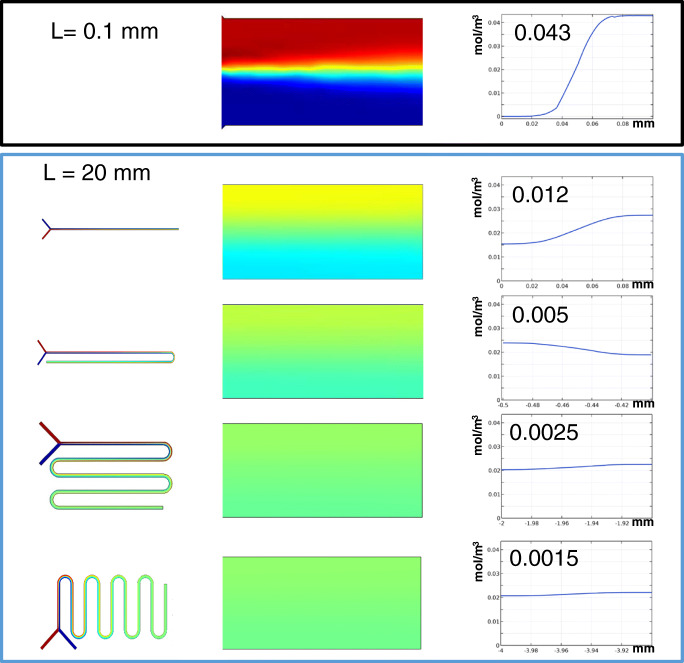
COMSOL simulations on the effect of incorporating several curves in the microchannel. The top panel shows the initial concentration gradient at *L* = 0.1 mm for all cases. The panel below shows concentration distributions at the end of the channel for four different cases: straight channel and channel including one, four, and eight curves. Plots on the right indicate the concentration distribution along the cross section at positions shown on the left (*L* = 0.1 mm and *L* = 20 mm). Water and albumin were introduced at the inlet at 1:1 ratio. Four channel segments were magnified for better visibility. Values at the bottom mark the distance from the entry point. (*L*, 2 cm; *D*_albumin_ = 6.1.10–11 m2/s; *v*, 3 mm/s)

**Fig. 3:**
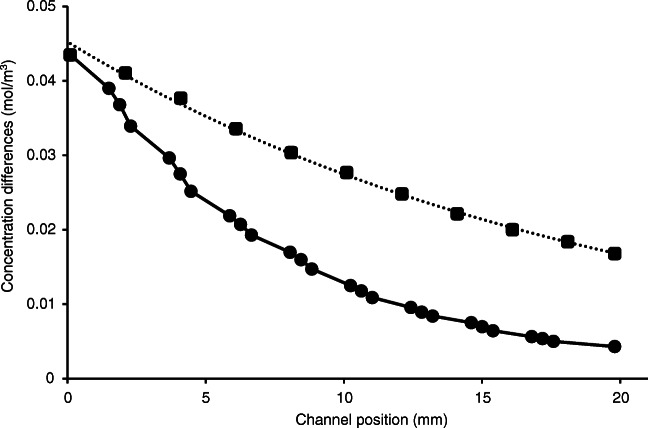
The gradual change in concentration distribution at different positions (length) of the channel containing 8 curves. The concentration differences were calculated based on cut lines of simulations, which were positioned before, in the middle, and after the curves (similarly as in Fig. ESM-7). The dotted line indicates the concentration differences in the case of a straight channel. Parameters were the same as in Fig. 2, except *v*, 10 mm/s

Because the curves support (even if slightly) the mixing of solutions in the channel, it is worth including several curves in the channel pattern. (On the other hand, the application of curves (serpentines) is inevitable if a long channel is to be arranged in a small-area microchip.) Figure 2 shows the concentration distributions at the outlet end of the channel (20 mm length) for four different cases: straight channel and channel including one, four, and eight curves. The effect the increasing number of curves have on the mixing is apparent. While in a straight channel the radial concentration difference was reduced from the initial 0.043 mM to 0.012 mM, in the channels including one, four, or eight curves the corresponding concentration differences were found to be 0.005 mM, 0.0025 mM, and 0.0015 mM. That is, with eight curves, an almost complete (97%) mixing could be achieved in the system studied, but in a straight channel, the mixing was only 72%. The contribution of the curves to the change in the concentration distribution at different positions (length) of the channel is gradually decreasing, i.e. the first curve has the largest relative mixing effect (Fig. 3). The 8th curve has only around 10% contribution to the mixing compared to the first curve. This means that, theoretically, the number of the curves in an IMER may not necessarily be increased beyond 10 in order to enhance the mixing effect; nevertheless, a longer channel can be formed in a microchip only in a serpentine shape. The “breaking points” in the diagram correspond to the position of the curvatures indicating a more sudden change in concentration difference, compared to the respective lengths of the straight channel.

### Application of the microreactor

**Fig. 4:**
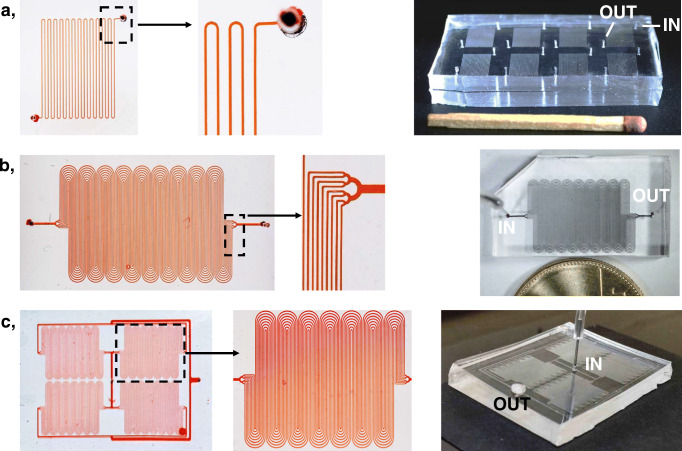
Images of three different microfluidic chips (on the right). The channel system of each microchip was filled with red food dye; certain sections were magnified for better visibility. Channel parameters: *w*, 25 μm; *L*, 20 cm; number of channels, 1, 8, and 32 for design A (**a**), B (**b**), and C (**c**), respectively

Based on the conclusions above, several μ-IMERs were designed and fabricated. In these chips, 25–75 μm channel widths were used and the height and the length of the channels were 25 μm and 200 mm, respectively. Because the channel lengths of these chips were 10 times longer than in the simulations, in cases where complete mixing was found in the simulations, presumably all proteins could reach the wall. In a standard-size (25 × 75 mm) microfluidic chip, 8 identical reactors can be arranged in a way that the reactors are properly far from each other to be able to easily operate even the neighbouring reactors in parallel (Fig. 4a). This pattern includes 24 curves. The peristaltic pump’s tubing can be connected to either port (inlet) of the channel. Because the PDMS is strongly hydrophobic, the digested solution of 10 μL volume appeared at the outlet as a hemisphere-shaped droplet, which can be easily drawn up with a micropipette.

In order to reduce the applicable flow rate in the channel (hence increase the residence time of the component), the liquid flow was split to 8 parallel streams at the inlet of the reactor and the liquid passed through the 8 parallel channels, which were merged at the outlet (Fig. 4b). In this design, the overall volumetric flow rate was identical to that of the previous design, but the rate could be reduced by a factor of 8 in each channel reactor (if the same pressure is applied). A further factor of 4 can be achieved in lowering the flow rate in the channel, if four identical reactor units including 8–8 parallel channel reactors (shown in Fig. 4b) are connected. In this arrangement, the sample is introduced in the centre part of the chip and it is split first to 4 parts and then to additional 8–8 parts (Fig. 4c).

The efficiency of the enzyme reactors of different designs was studied by digesting human saliva samples. Saliva is a mixture of different proteins and other biological compounds. Fig. ESM-9 shows the electropherograms of human saliva digested via standard in-solution procedure (16 h, 37 °C) and on-chip using a microchip (design B in Fig. 4b; channel width, 75 μm; 2 min contact time; 25 °C). On the electropherograms, a large number of peaks with similar migration times were obtained. Although the signal intensities for the corresponding peaks differ, this probably only means that the enzymes immobilized and being present freely in solution cleave a given bond with different probability. Because the number of the components obtained in the digested sample is similar (55–62) and no residues of undigested proteins were detected, the developed enzyme reactor can be considered useful for peptide mapping. Very similar digestions were obtained with the other two microchip designs.

**Fig. 5:**
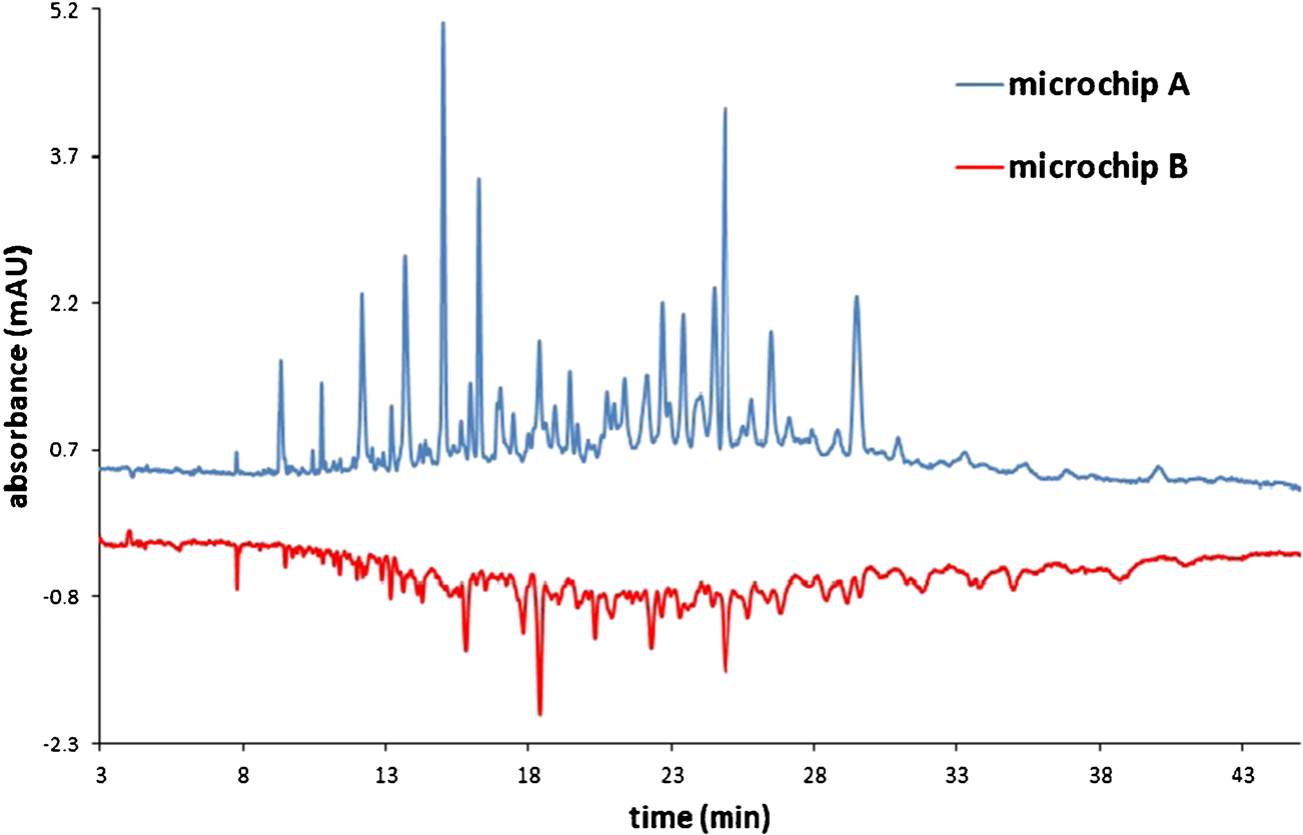
Comparison of peptide maps obtained from reactor-based digestions using microchip A (design B; *w*, 25 μm) and microchip B (design B; *w*, 75 μm). CE conditions: fused silica capillary; i.d., 50 μm; *L*_eff_, 71.5 cm; BGE, 100 mM PB (pH, 2.2); *U*, 30 kV; sample introduction, 50 mbar, 10 s; *λ*, 200 nm

The repeatability of the migration times and peak areas of the components in the capillary electrophoretic measurements of the digested sample was better than 1.2 and 7.8 RSD%, respectively, based on the six consecutive measurements indicated in Fig. ESM-10 (migration times corrected with two time reference points). For the reproducibility study of on-chip digestion, the same chip layout (design B; width, 25 μm) but five distinct reactors were used. The RSD% data calculated for two randomly selected peaks in the five electropherograms were 0.88 and 1.05% for migration times and 36.1 and 20.2% for peak areas, respectively. In Fig ESM-11, a butterfly plot was generated choosing those two peptide maps that differed from each other the most. Since nearly all peaks of the two electropherograms can be almost perfectly mirrored, even a simple visual observation suggests a very good correlation between the peak patterns. The peak patterns were relatively similar when microchips differing only in channel width (25 and 75 μm, reactor design B) were used (Fig. 5). The RSD% data calculated for five randomly selected peaks in the five electropherograms were better than 1.32 RSD% for migration times. A larger number of components could be found for the wider microreactor (41 and 55 for chips with a channel width of 25 μm and 75 μm, respectively), and the intensities of the peaks were smaller with the wider reactor. It is assumed that the longer contact time provided increased probability for a cleavage to occur, which led to a higher number of components and smaller peak intensities for the microreactor with a 75-μm width. Although in these experiments the application of the 75-μm-wide channel seemed more efficient, if the same linear speed (thus the same contact time) using different pumping pressures was applied in the microchips with channel widths of 25 and 75 μm, no remarkable differences were obtained in peak patterns.

**Fig. 6:**
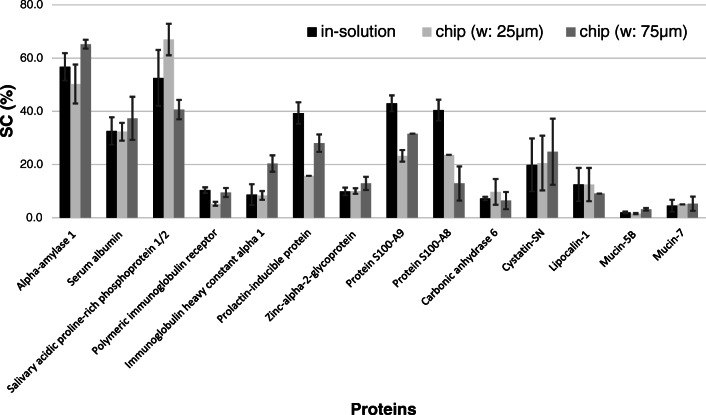
Sequence coverage (SC%) values of the proteins identified in human saliva by CE-MS/MS using in-solution and on-chip digestions. Only 14 proteins identified with at least two unique peptides are shown

The efficiency of protein digestion can be characterized by the sequence coverage (SC%, that is the % of the protein detected as peptides) as well. SC% of the proteins determined in human saliva by CE-MS/MS using in-solution and on-chip digestions with two different channel widths were compared in Fig. 6 and Table ESM-1 (only 14 proteins identified with at least two unique peptides are shown). The obtained SC% values were in the same range for the three kinds of digestions. For 3 proteins, the SC% values were somewhat higher for in-solution digestion compared to the chip-based digestion; however, for the remaining 11 proteins, the chips showed a modest improvement in performance over the solution-based digestion. In the case of on-chip digestions, the chip with 75 μm width provided slightly higher SC% values for 10 proteins and only for 4 proteins did a channel width of 25 μm give higher SC%. These results agree with the above conclusion: the use of the microreactor capable of providing longer contact time between the protein substrate and the immobilized trypsin led to a more efficient digestion.

For the reproducibility study of on-chip digestion, 5–5 consecutive digestions were carried out for all three platforms (in-solution and chip design B (*w*_1_, 25 μm; *w*_2_, 75 μm)). In the case of chip-based digestions, the 5–5 digestions were performed using different reactors (for the evaluation of the reliability of enzyme immobilization). The precision of each digestion strategy was evaluated in terms of the scattering of SC% values (Fig. 6), which ranged between 0 and 39%.

## Conclusions

The goal of this paper was to study the design, efficiency, and applicability of a simple PDMS microfluidic chip used for rapid protein digestion. PDMS is a material frequently preferred for the fabrication of microfluidic chips; its superior adsorptive ability has recently been utilized for nonspecific adsorption of enzymes in order to prepare enzymatic microreactors. In our previous work [25], it was shown that the wall of the empty, long channel system in a PDMS chip can be used directly as a solid support for trypsin immobilization and thus to develop IMERs with simple open channel geometry. There have been numerous impressive IMER constructions published so far, most of which enable the efficient or complete proteolysis of the substrates of interest. The significance of this particular IMER setup lies in its simplicity; the immobilization procedure only consists of trypsin solution being transported through the native PDMS microchannels, excluding laborious, multi-step procedures, where each step might entail sources of error. It is obvious, however, that such IMERs do not exhibit the lifetime and enzyme loading capacity characteristic of other published IMERs which utilize monolithic or packed-bed microchannels or even porous layer open tubular reactors. The limitations in the longevity of the IMER are resolved by the straightforward and fast (~ 10 min) immobilization process. Furthermore, by appropriately manipulating the liquid flow in wall-coated μ-IMERs, it is possible to mitigate the problem of relatively low enzyme load and diffusion-limited mass transfer in order to achieve successful proteolytic cleavage. Our objective was to study such open-channel geometries in a layer-bed-type immobilized enzyme reactor system with computer-aided simulations. The COMSOL simulation software proved to be a useful tool for designing and optimizing the channel pattern and for giving us an insight into the effects the channel width, channel length, and curves as well as flow rates have on the radial diffusion and mixing in the channel. The simulations obtained for the different channel designs and configurations well supported the identification of the relevant aspects that have to be taken into account for achieving optimal conditions for improving the performance of the microfluidic IMERs and allow us a better understanding of laminar liquid flow in empty channel geometries.

*S*/*V* values largely increase with the narrowing of the empty channel; however, two notable drawbacks discourage us from endorsing such narrow channels (*w* ≤ 10 μm): (i) the lack of sophisticated microfluidic facilities in an average laboratory hampers the successful fabrication of these small features and (ii) the transportation of a 10-μL-volume sample solution through the reactor can be unreasonably prolonged (~ 10 h). The duration of transportation might be significantly accelerated (~ 3.5 s) using 100-μm-wide channels, albeit at the cost of decreased digestion efficiency, since thorough mixing in the channel is inhibited due to the laminar flow. Therefore, the recommended range of the channel width was found to be 25–75 μm. The simulation clearly showed that curves support (slightly) the mixing of solutions in the channel even in the strong laminar flow conditions (Re < 1); thus, it is worth including several curves in the channel system.

In our previous work [22], we integrated micropillars into the channel in order to increase the *S*/*V* ratio and the total surface (*S*) of the reactor. The use of empty channels instead of the array of pillars can be considered as a simplification, but our goal and the key point of the developments of IMERs was to study whether the *S*/*V* ratio of an empty channel pattern can be increased to the degree of the pillars’ pattern. In the case of a 25-μm-wide empty channel, an *S*/*V* ratio similar to the channels containing 25-μm pillars and 25-μm interpillar distance can be achieved, but the total surface is much smaller in an empty channel. In the present study, it has been shown that both the *S*/*V* and the *S* can be similarly high if multiple empty channels are applied (split and merged between the inlet and outlet ports, respectively). With the proposed empty channel reactor (*w*, 25 μm; *L*, 20 cm; number of channels, 8), complete digestion of the proteins can be achieved just like with the reactor including pillars or with the classical in-solution method. The *S*/*V*-increasing effect of the pillar arrangement is only significant if the channel is tightly “packed”. However, technical difficulties (e.g. light scatter during photoresist exposure or pattern collapse due to PDMS flexibility [44]) pose limitations on pillar density. Precise and refined micropillar patterning onto harder materials can be carried out utilizing other, more sophisticated technologies, but such processes are generally time consuming and more expensive. On the other hand, the proposed serpentine-like channels are considerably easier to prepare and the split-flow architecture ensures a sufficiently high total surface. Another advantage of the empty channels is that the hydrodynamics in the empty channel could be more easily monitored and also tuned by the application of curves (with proper numbers and arch dimension). The proposed μ-IMER provided similar efficiencies for the digestion of saliva as the standard in-solution digestion procedure. We have shown that the flow rate and channel geometry play a crucial role in achieving efficient proteolysis. Split-flow structures are especially advantageous because of the increased contact time, despite the overall higher volumetric flow rate. Based on our investigations (both theoretical and experimental), it can be concluded that in the 25–75-μm range, no specific value for channel width can be selected as the gold standard, since the desired digestion efficiency can be attained by fine-tuning flow conditions and channel parameters.

## Supplementary information

ESM 1 (PPTX 4927 kb)

## Declarations

Informed consent for saliva sampling was obtained from the healthy female volunteer included in the study. The procedure performed involving the volunteer was in accordance with the ethical standards of the institutional research committee (Regional Institutional Research Ethics Committee, Clinical Center, University of Debrecen).
