# Supplementary material for: Study of the geometry of open channels in a layer-bed-type microfluidic immobilized enzyme reactor
Source: Anal Bioanal Chem. 2021 Aug 10;413(25):6321–32. doi: 10.1007/s00216-021-03588-x (PMC8487885; doi:10.1007/s00216-021-03588-x)
Supplement: Supplementary file 1 — (PPTX 4927 kb) [file 216_2021_3588_MOESM1_ESM.pptx]

## Slide 1
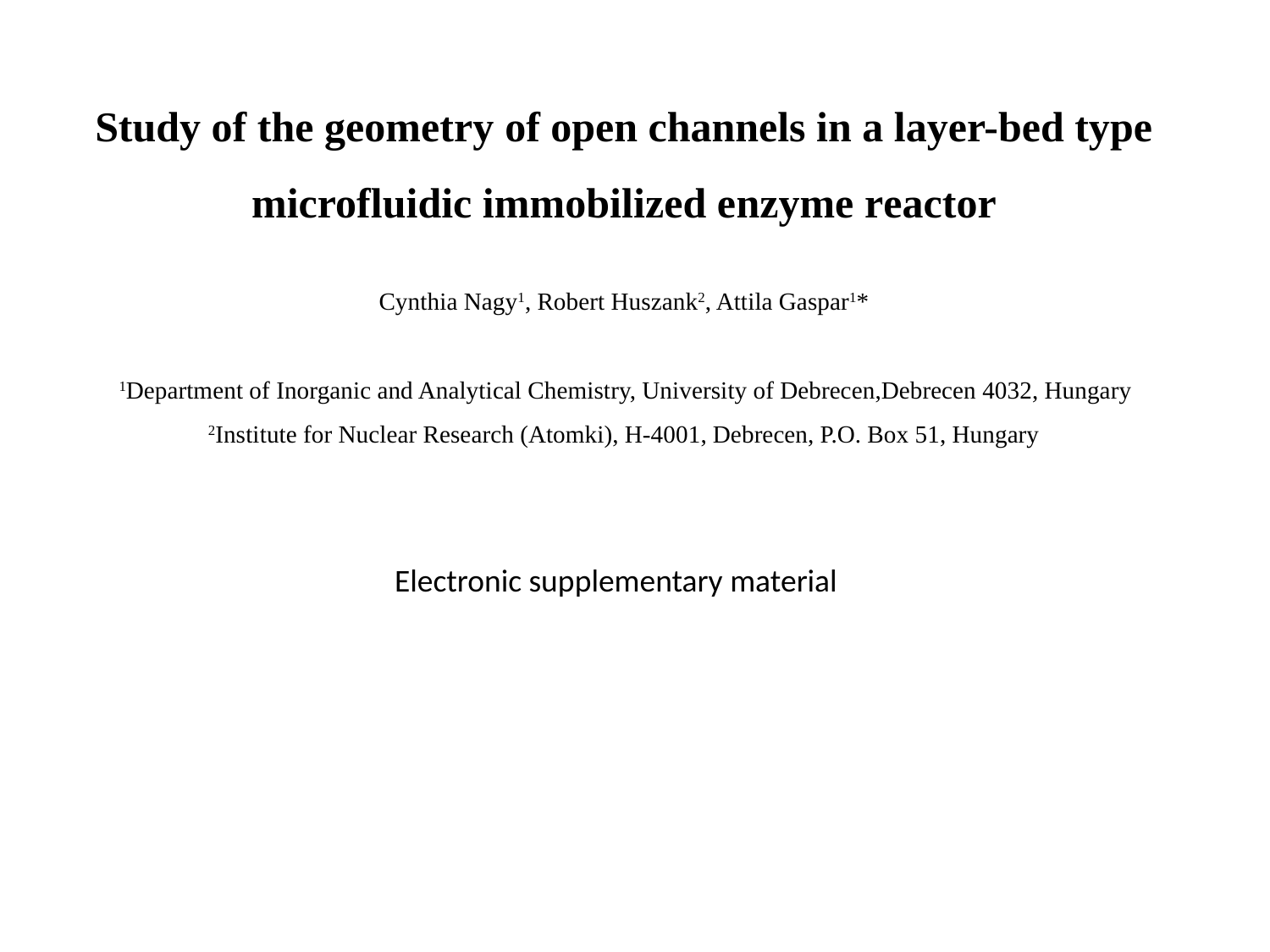

Study of the geometry of open channels in a layer-bed type microfluidic immobilized enzyme reactor
Cynthia Nagy1, Robert Huszank2, Attila Gaspar1*
 1Department of Inorganic and Analytical Chemistry, University of Debrecen,Debrecen 4032, Hungary
2Institute for Nuclear Research (Atomki), H-4001, Debrecen, P.O. Box 51, Hungary
Electronic supplementary material

## Slide 2
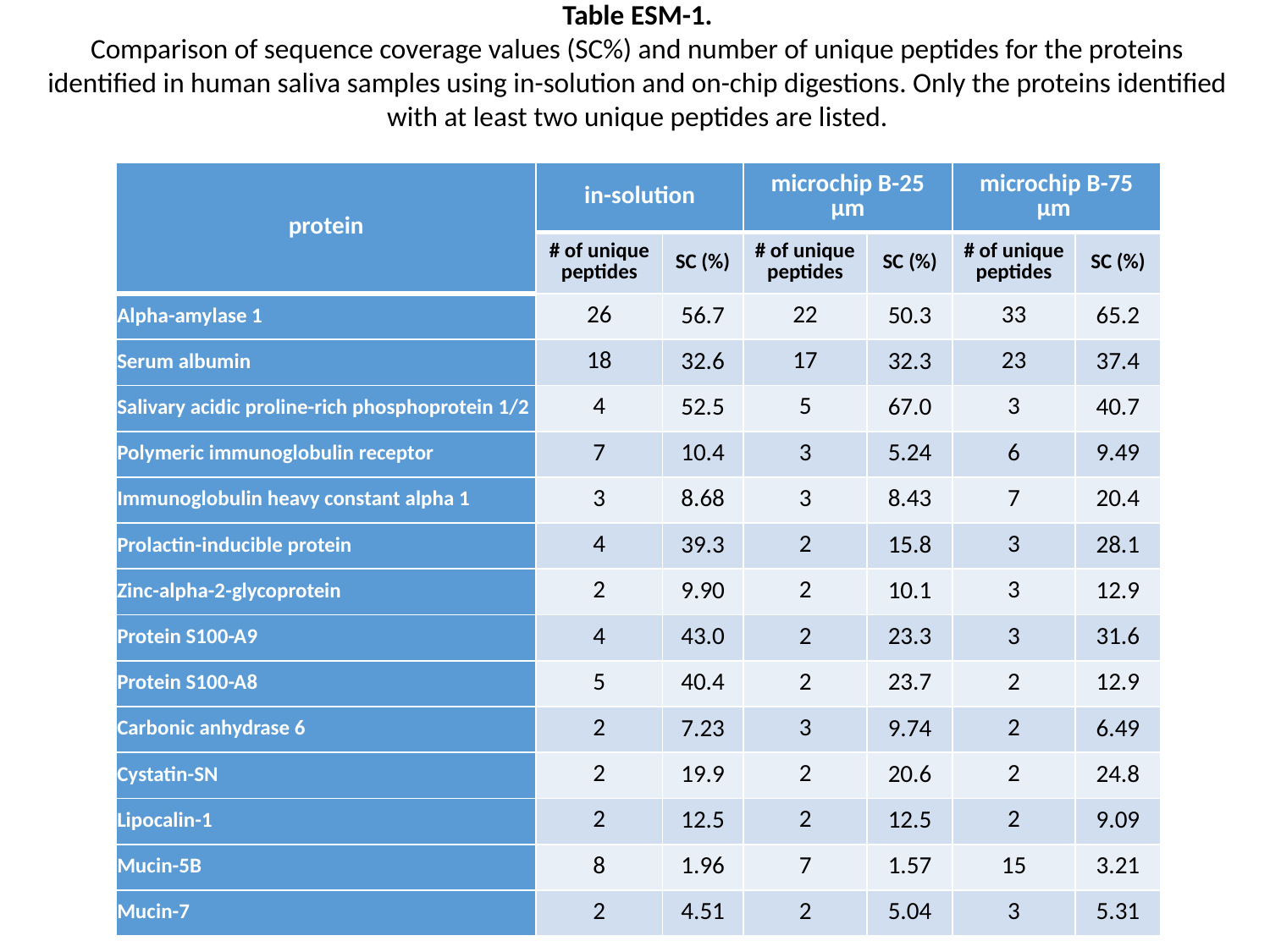

Table ESM-1.
Comparison of sequence coverage values (SC%) and number of unique peptides for the proteins identified in human saliva samples using in-solution and on-chip digestions. Only the proteins identified with at least two unique peptides are listed.
| protein | in-solution | | microchip B-25 µm | | microchip B-75 µm | |
| --- | --- | --- | --- | --- | --- | --- |
| | # of unique peptides | SC (%) | # of unique peptides | SC (%) | # of unique peptides | SC (%) |
| Alpha-amylase 1 | 26 | 56.7 | 22 | 50.3 | 33 | 65.2 |
| Serum albumin | 18 | 32.6 | 17 | 32.3 | 23 | 37.4 |
| Salivary acidic proline-rich phosphoprotein 1/2 | 4 | 52.5 | 5 | 67.0 | 3 | 40.7 |
| Polymeric immunoglobulin receptor | 7 | 10.4 | 3 | 5.24 | 6 | 9.49 |
| Immunoglobulin heavy constant alpha 1 | 3 | 8.68 | 3 | 8.43 | 7 | 20.4 |
| Prolactin-inducible protein | 4 | 39.3 | 2 | 15.8 | 3 | 28.1 |
| Zinc-alpha-2-glycoprotein | 2 | 9.90 | 2 | 10.1 | 3 | 12.9 |
| Protein S100-A9 | 4 | 43.0 | 2 | 23.3 | 3 | 31.6 |
| Protein S100-A8 | 5 | 40.4 | 2 | 23.7 | 2 | 12.9 |
| Carbonic anhydrase 6 | 2 | 7.23 | 3 | 9.74 | 2 | 6.49 |
| Cystatin-SN | 2 | 19.9 | 2 | 20.6 | 2 | 24.8 |
| Lipocalin-1 | 2 | 12.5 | 2 | 12.5 | 2 | 9.09 |
| Mucin-5B | 8 | 1.96 | 7 | 1.57 | 15 | 3.21 |
| Mucin-7 | 2 | 4.51 | 2 | 5.04 | 3 | 5.31 |

## Slide 3
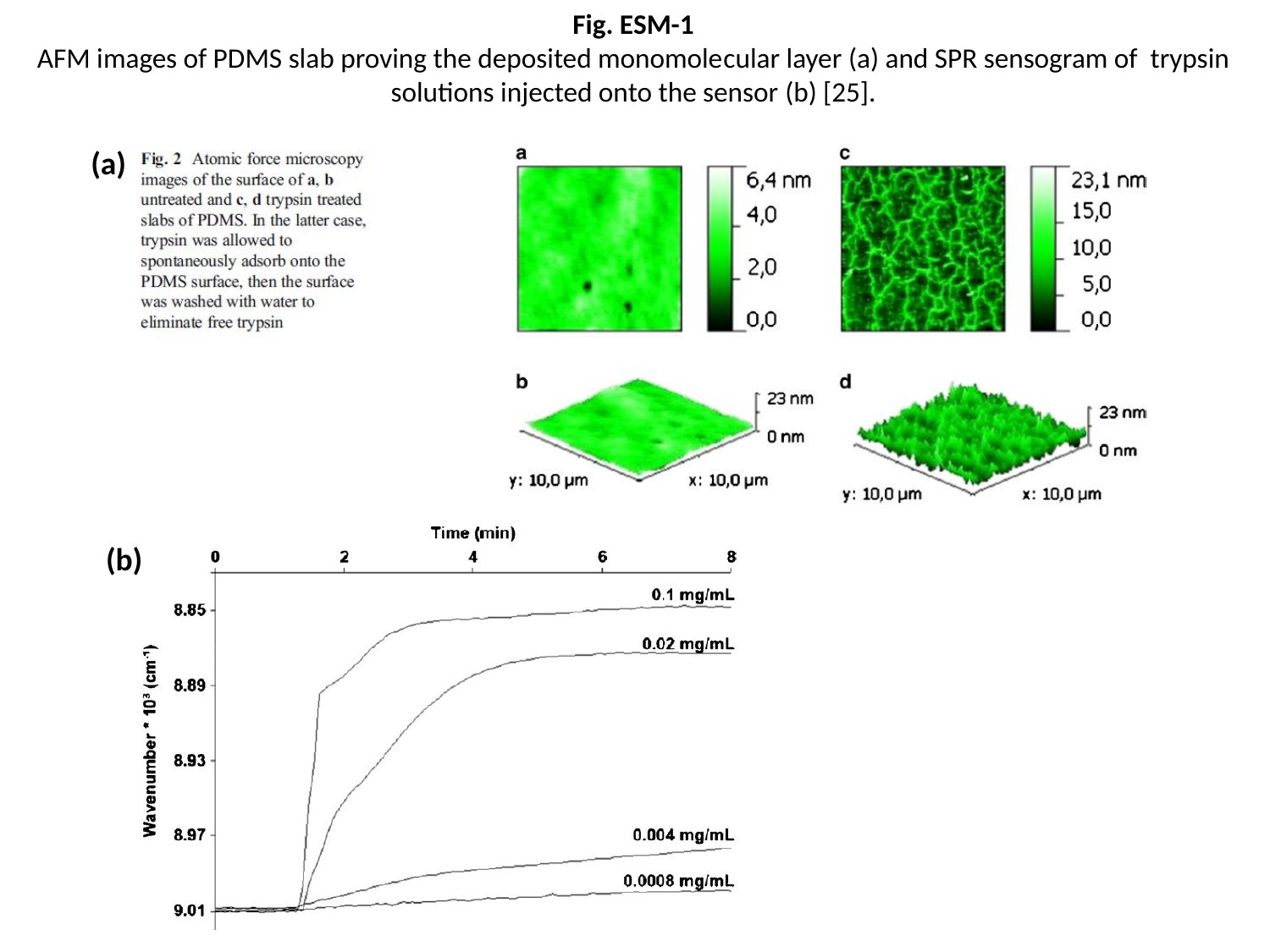

Fig. ESM-1
AFM images of PDMS slab proving the deposited monomolecular layer (a) and SPR sensogram of trypsin solutions injected onto the sensor (b) [25].
a,
(a)
b,
(b)
c,

## Slide 4
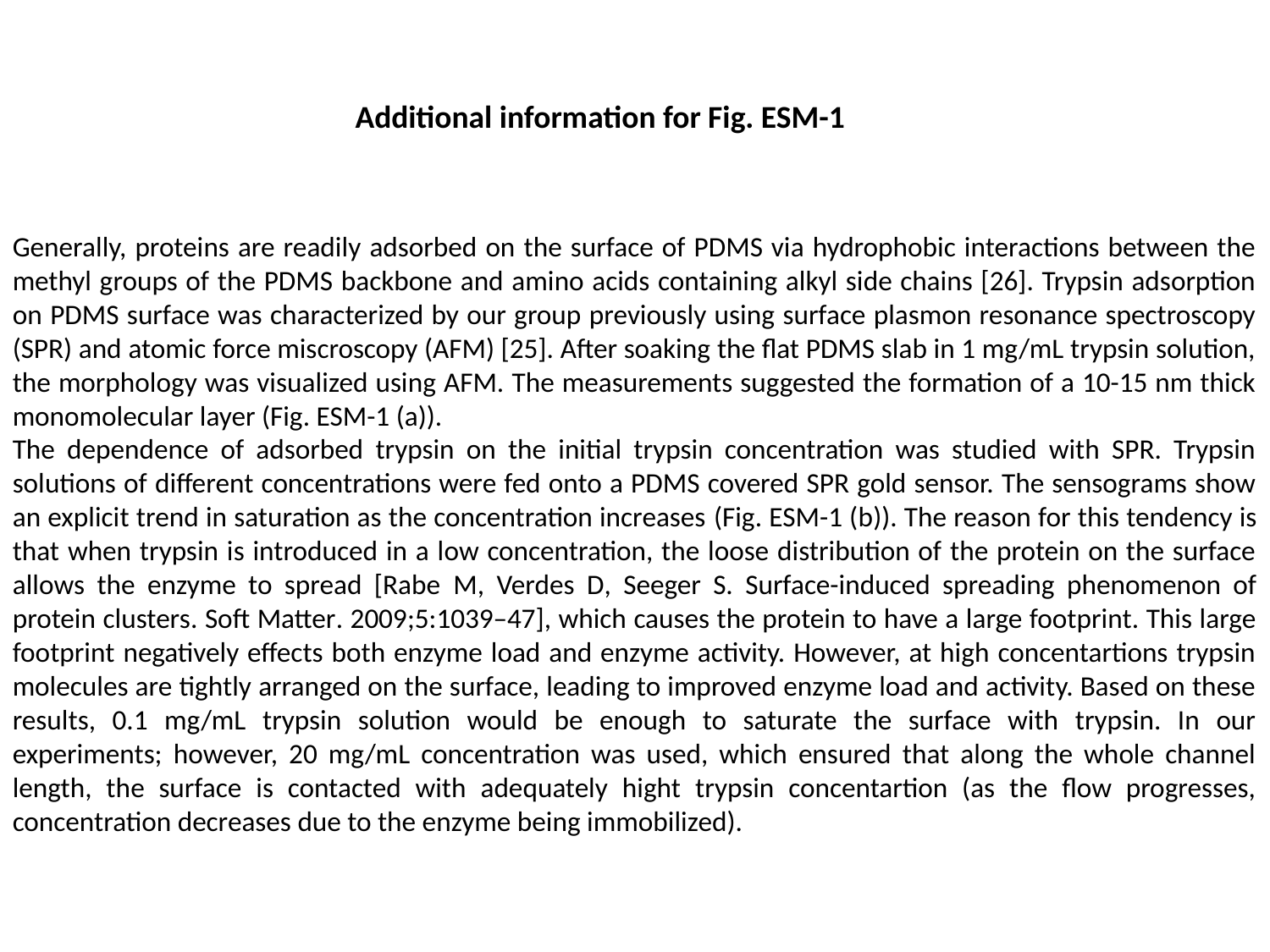

Additional information for Fig. ESM-1
a,
Generally, proteins are readily adsorbed on the surface of PDMS via hydrophobic interactions between the methyl groups of the PDMS backbone and amino acids containing alkyl side chains [26]. Trypsin adsorption on PDMS surface was characterized by our group previously using surface plasmon resonance spectroscopy (SPR) and atomic force miscroscopy (AFM) [25]. After soaking the flat PDMS slab in 1 mg/mL trypsin solution, the morphology was visualized using AFM. The measurements suggested the formation of a 10-15 nm thick monomolecular layer (Fig. ESM-1 (a)).
The dependence of adsorbed trypsin on the initial trypsin concentration was studied with SPR. Trypsin solutions of different concentrations were fed onto a PDMS covered SPR gold sensor. The sensograms show an explicit trend in saturation as the concentration increases (Fig. ESM-1 (b)). The reason for this tendency is that when trypsin is introduced in a low concentration, the loose distribution of the protein on the surface allows the enzyme to spread [Rabe M, Verdes D, Seeger S. Surface-induced spreading phenomenon of protein clusters. Soft Matter. 2009;5:1039–47], which causes the protein to have a large footprint. This large footprint negatively effects both enzyme load and enzyme activity. However, at high concentartions trypsin molecules are tightly arranged on the surface, leading to improved enzyme load and activity. Based on these results, 0.1 mg/mL trypsin solution would be enough to saturate the surface with trypsin. In our experiments; however, 20 mg/mL concentration was used, which ensured that along the whole channel length, the surface is contacted with adequately hight trypsin concentartion (as the flow progresses, concentration decreases due to the enzyme being immobilized).
b,
c,

## Slide 5
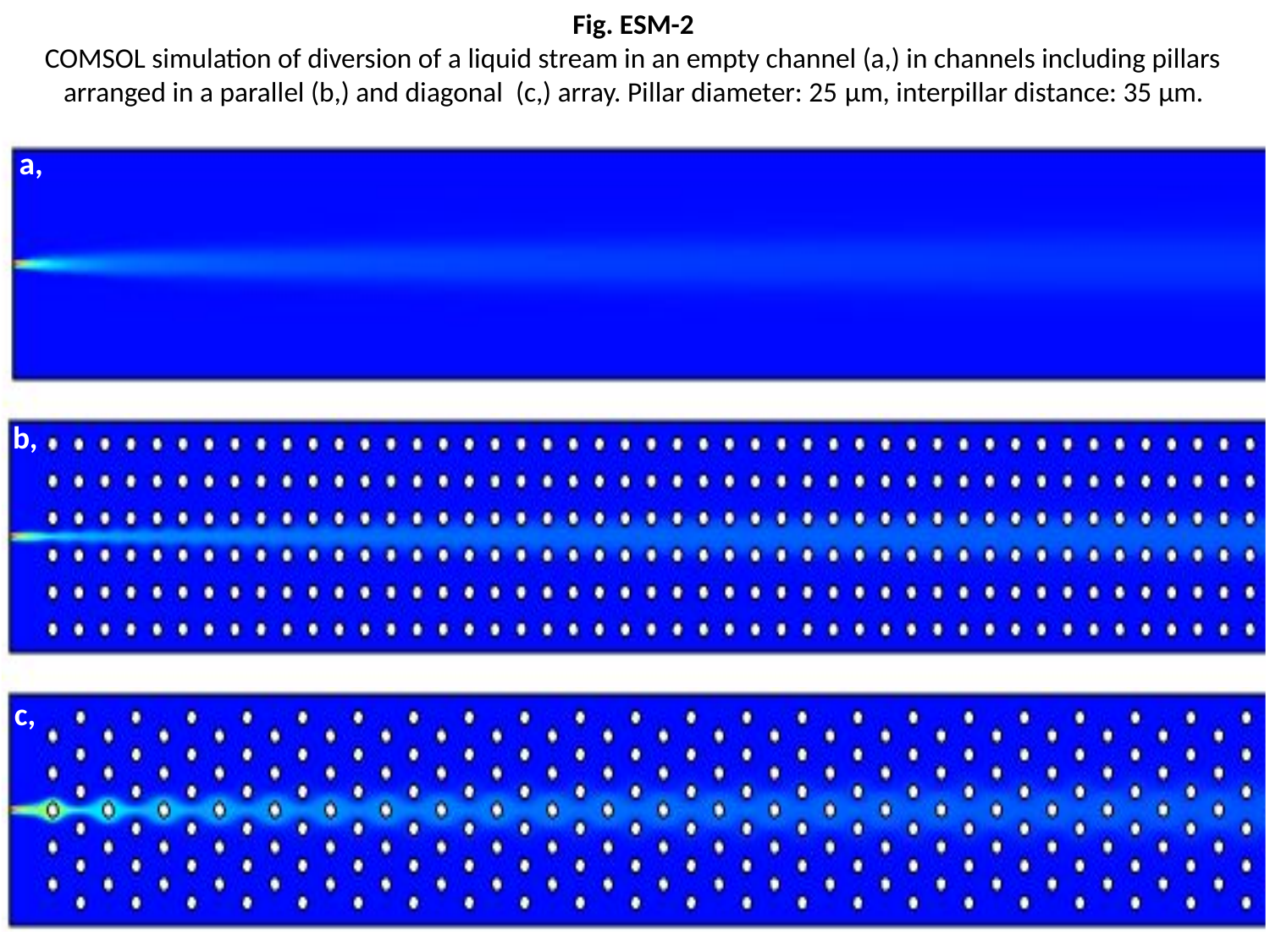

Fig. ESM-2
COMSOL simulation of diversion of a liquid stream in an empty channel (a,) in channels including pillars arranged in a parallel (b,) and diagonal (c,) array. Pillar diameter: 25 μm, interpillar distance: 35 μm.
a,
b,
c,

## Slide 6
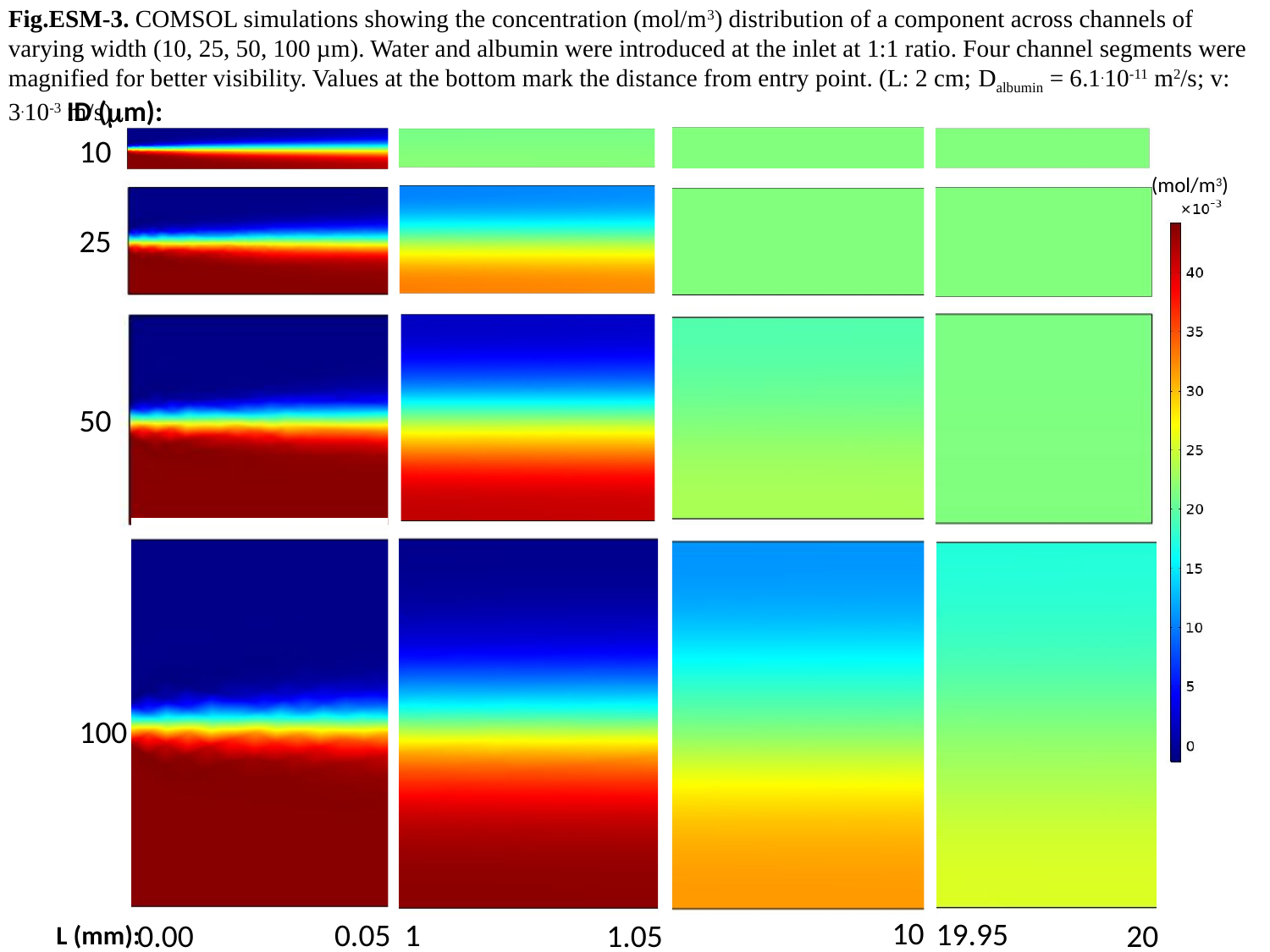

Fig.ESM-3. COMSOL simulations showing the concentration (mol/m3) distribution of a component across channels of varying width (10, 25, 50, 100 µm). Water and albumin were introduced at the inlet at 1:1 ratio. Four channel segments were magnified for better visibility. Values at the bottom mark the distance from entry point. (L: 2 cm; Dalbumin = 6.1.10-11 m2/s; v: 3.10-3 m/s)
ID (mm):
10
(mol/m3)
25
50
100
10
19.95
1
0.05
20
0.00
1.05
L (mm):

## Slide 7
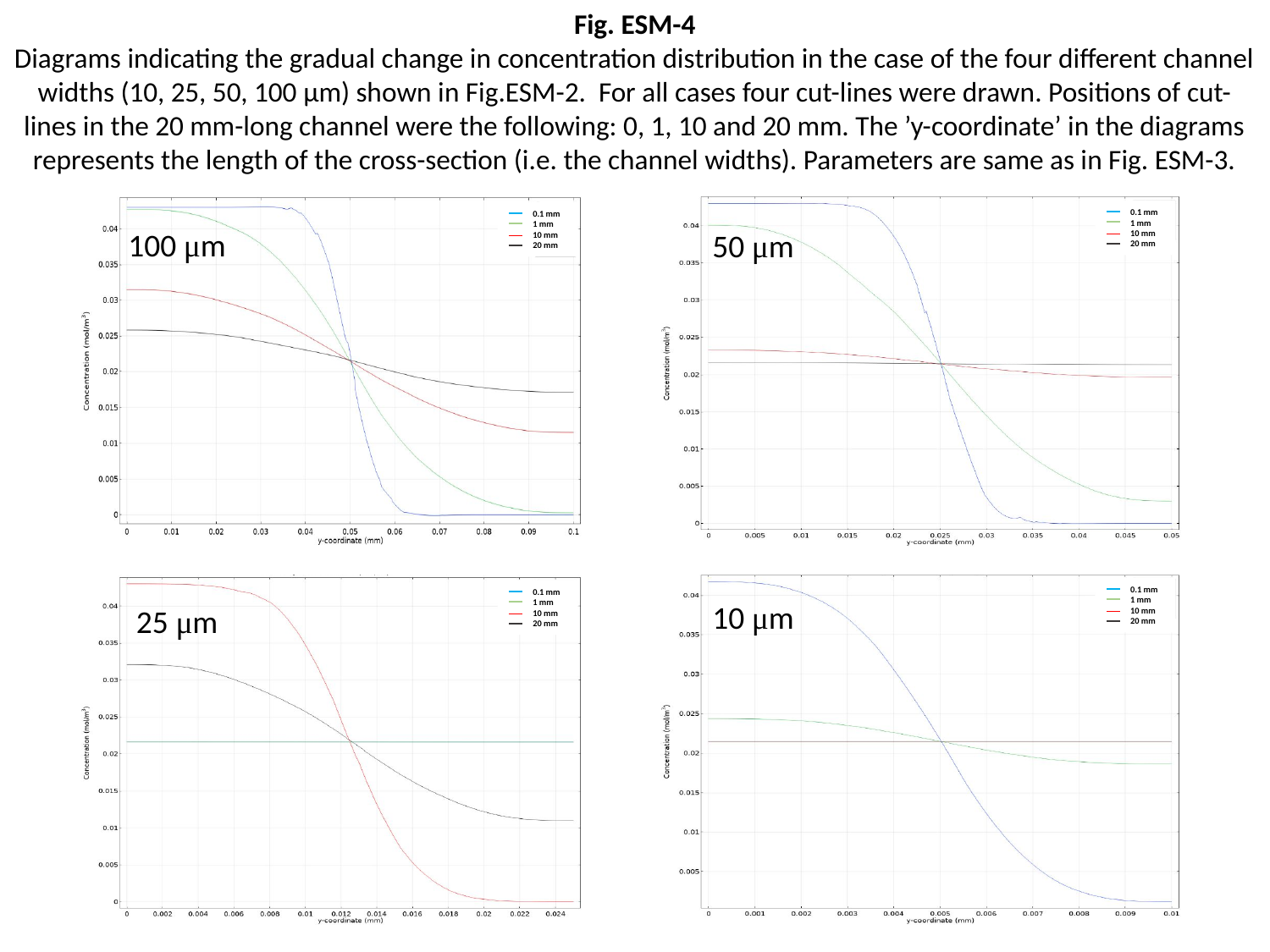

Fig. ESM-4
Diagrams indicating the gradual change in concentration distribution in the case of the four different channel widths (10, 25, 50, 100 μm) shown in Fig.ESM-2. For all cases four cut-lines were drawn. Positions of cut-lines in the 20 mm-long channel were the following: 0, 1, 10 and 20 mm. The ’y-coordinate’ in the diagrams represents the length of the cross-section (i.e. the channel widths). Parameters are same as in Fig. ESM-3.
 0.1 mm
 1 mm
 10 mm
 20 mm
 0.1 mm
 1 mm
 10 mm
 20 mm
100 μm
50 μm
 0.1 mm
 1 mm
 10 mm
 20 mm
 0.1 mm
 1 mm
 10 mm
 20 mm
10 μm
25 μm

## Slide 8
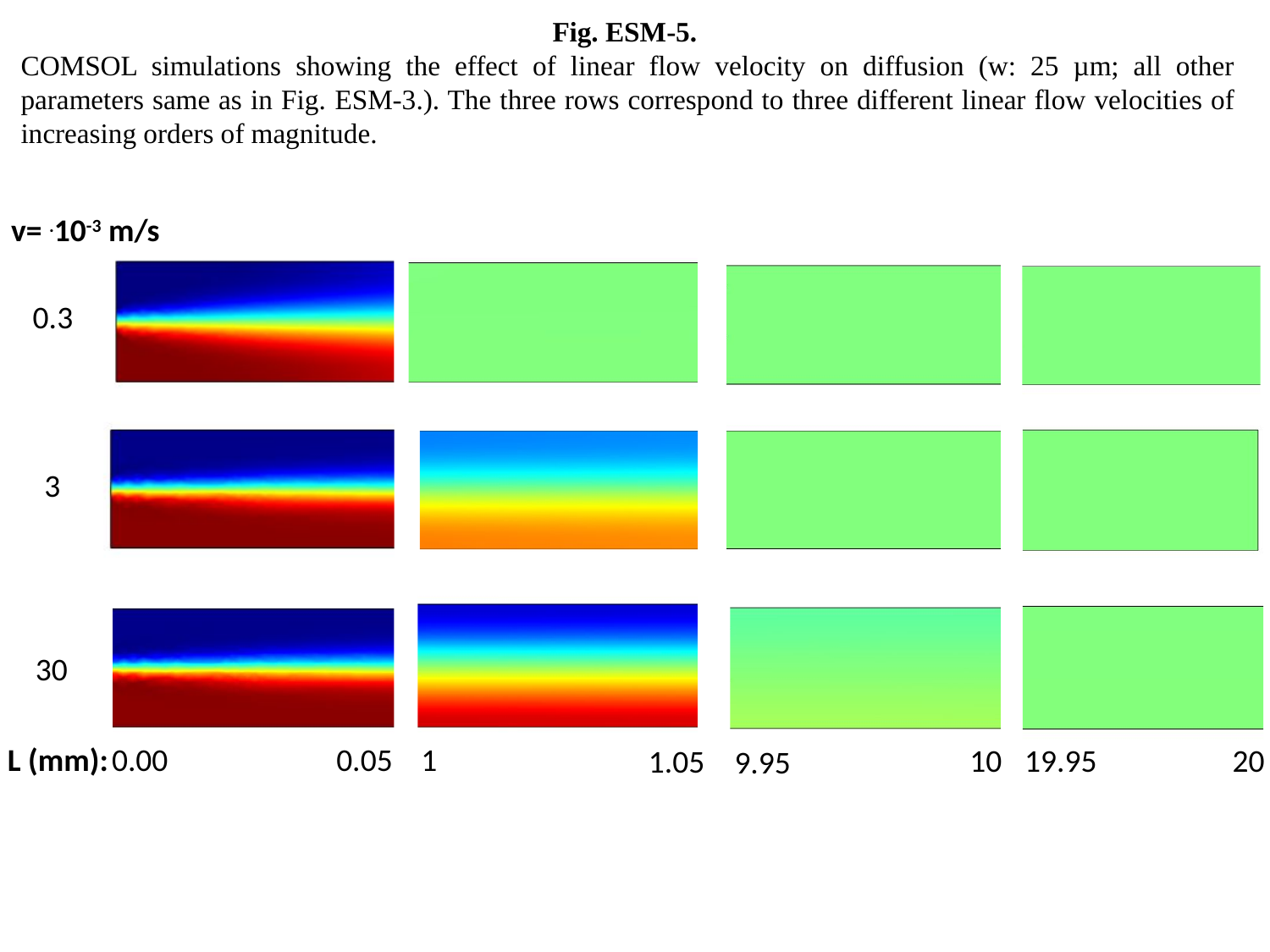

Fig. ESM-5.
COMSOL simulations showing the effect of linear flow velocity on diffusion (w: 25 µm; all other parameters same as in Fig. ESM-3.). The three rows correspond to three different linear flow velocities of increasing orders of magnitude.
v= .10-3 m/s
0.3
3
30
0.00
1
0.05
L (mm):
10
20
19.95
1.05
9.95

## Slide 9
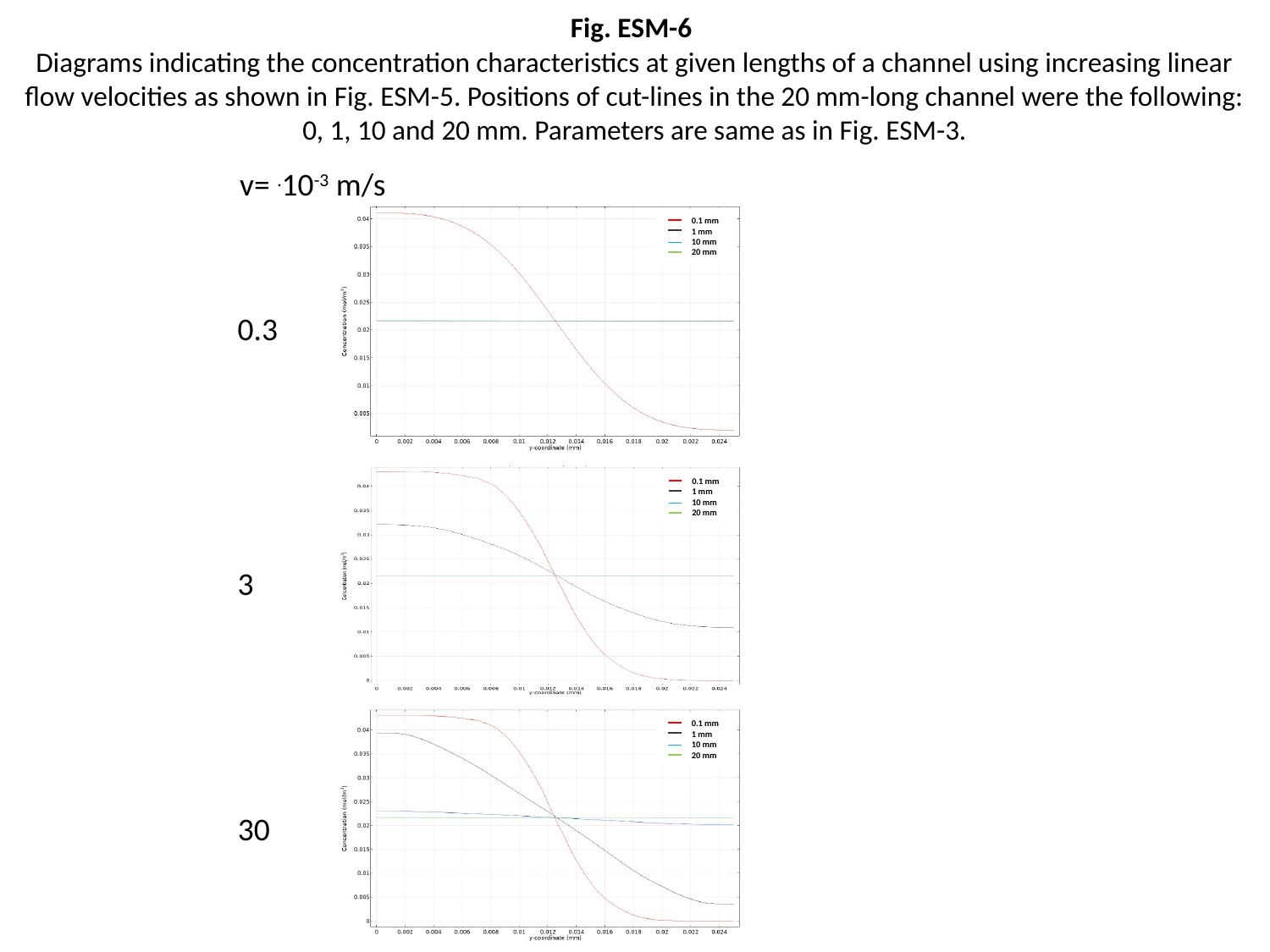

Fig. ESM-6
Diagrams indicating the concentration characteristics at given lengths of a channel using increasing linear flow velocities as shown in Fig. ESM-5. Positions of cut-lines in the 20 mm-long channel were the following: 0, 1, 10 and 20 mm. Parameters are same as in Fig. ESM-3.
v= .10-3 m/s
 0.1 mm
 1 mm
 10 mm
 20 mm
0.3
 0.1 mm
 1 mm
 10 mm
 20 mm
3
 0.1 mm
 1 mm
 10 mm
 20 mm
30

## Slide 10
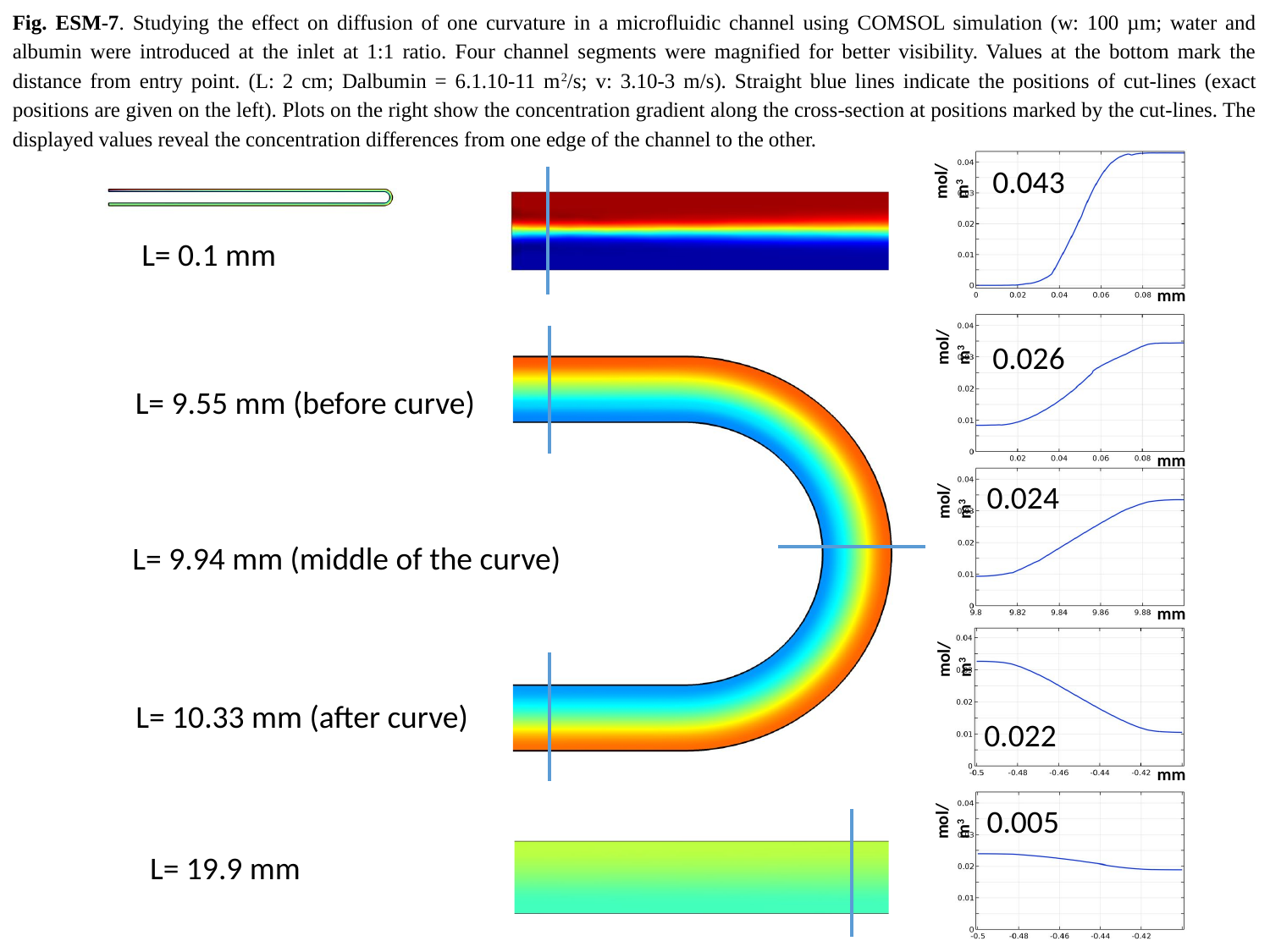

Fig. ESM-7. Studying the effect on diffusion of one curvature in a microfluidic channel using COMSOL simulation (w: 100 µm; water and albumin were introduced at the inlet at 1:1 ratio. Four channel segments were magnified for better visibility. Values at the bottom mark the distance from entry point. (L: 2 cm; Dalbumin = 6.1.10-11 m2/s; v: 3.10-3 m/s). Straight blue lines indicate the positions of cut-lines (exact positions are given on the left). Plots on the right show the concentration gradient along the cross-section at positions marked by the cut-lines. The displayed values reveal the concentration differences from one edge of the channel to the other.
mol/m3
0.043
L= 0.1 mm
mm
mol/m3
0.026
L= 9.55 mm (before curve)
mm
mol/m3
0.024
L= 9.94 mm (middle of the curve)
mm
mol/m3
L= 10.33 mm (after curve)
0.022
mm
mol/m3
0.005
L= 19.9 mm

## Slide 11
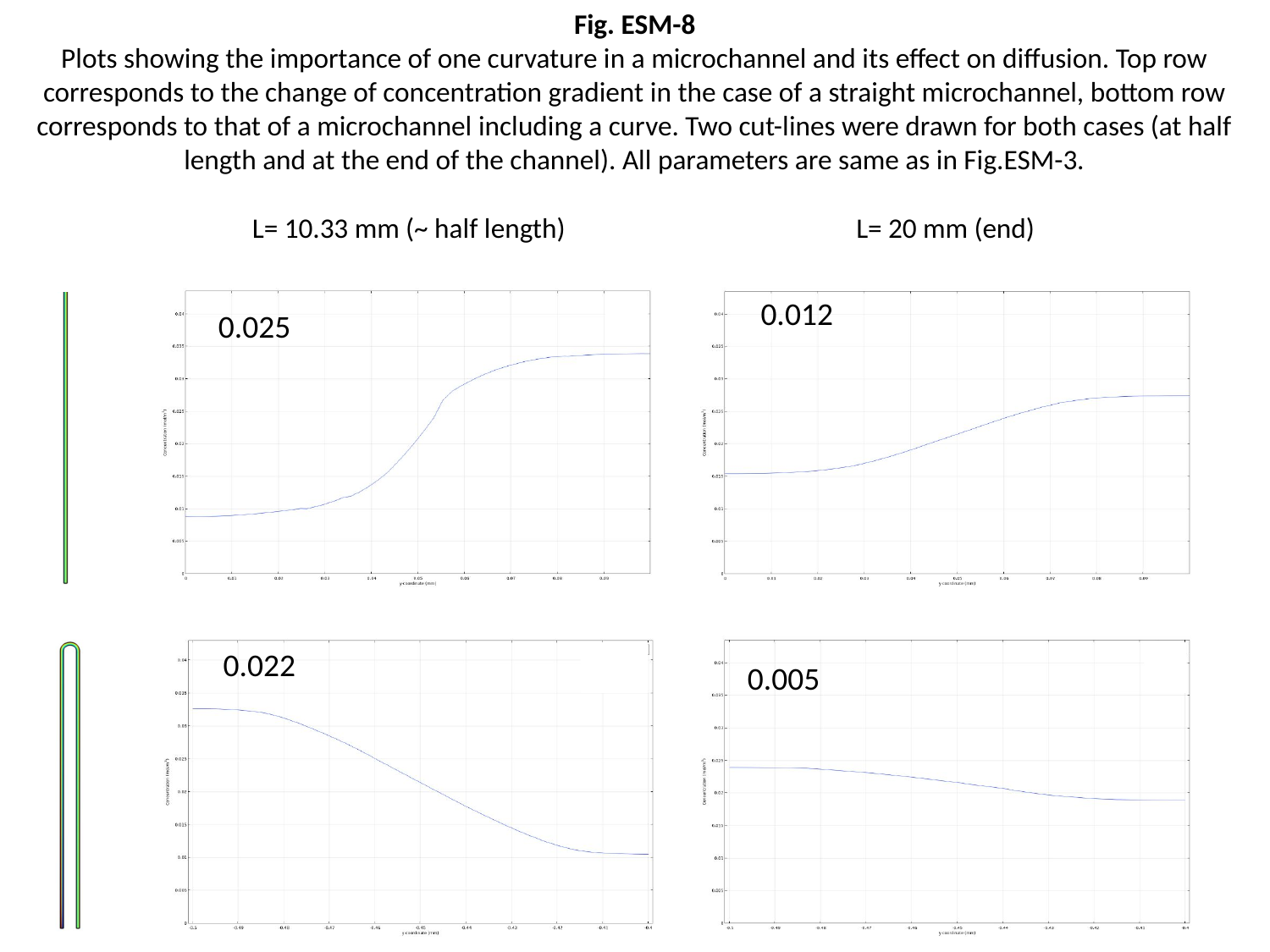

Fig. ESM-8
Plots showing the importance of one curvature in a microchannel and its effect on diffusion. Top row corresponds to the change of concentration gradient in the case of a straight microchannel, bottom row corresponds to that of a microchannel including a curve. Two cut-lines were drawn for both cases (at half length and at the end of the channel). All parameters are same as in Fig.ESM-3.
L= 10.33 mm (~ half length)
L= 20 mm (end)
0.012
0.025
0.022
0.005

## Slide 12
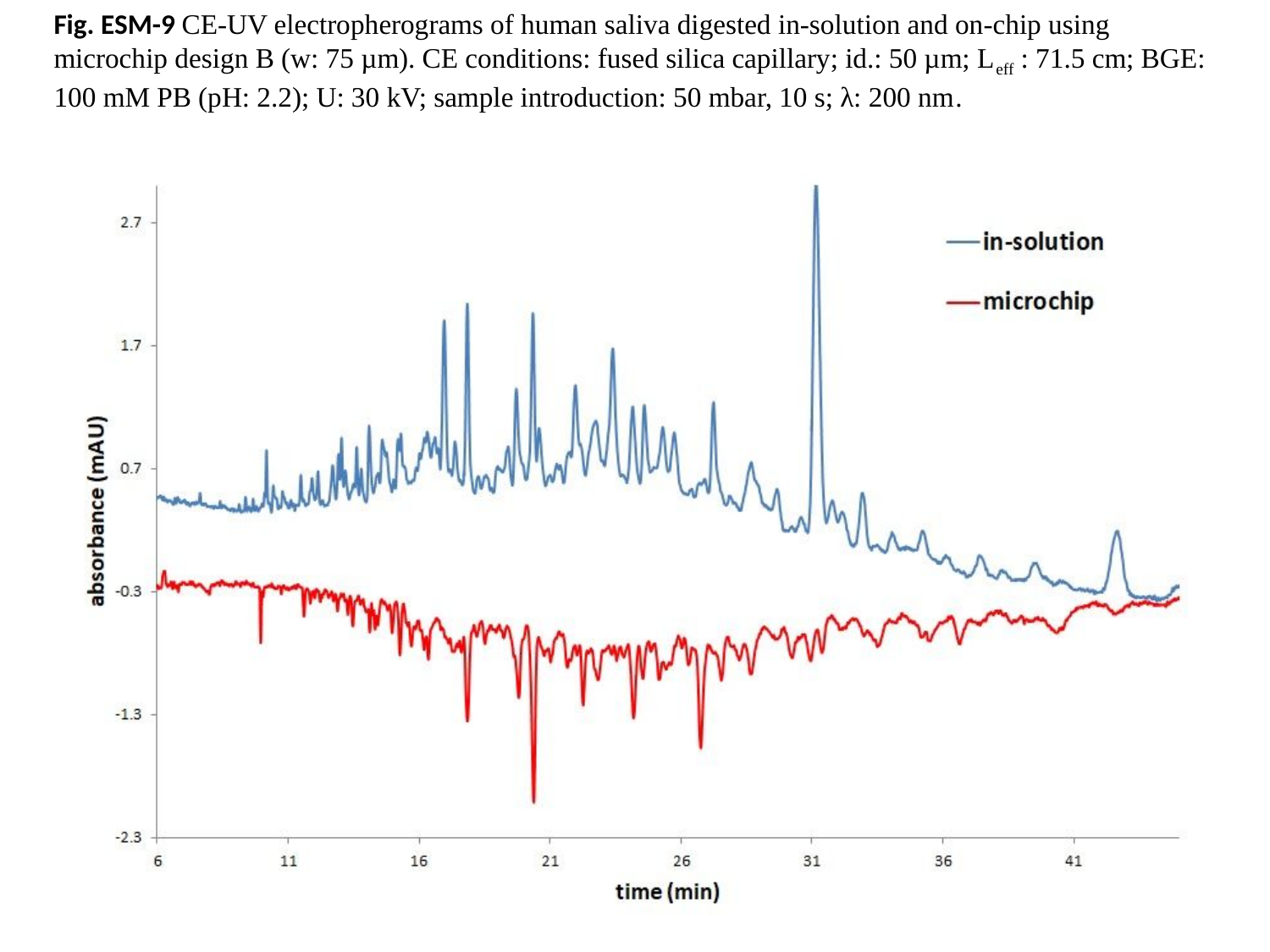

Fig. ESM-9 CE-UV electropherograms of human saliva digested in-solution and on-chip using microchip design B (w: 75 µm). CE conditions: fused silica capillary; id.: 50 µm; Leff : 71.5 cm; BGE: 100 mM PB (pH: 2.2); U: 30 kV; sample introduction: 50 mbar, 10 s; λ: 200 nm.

## Slide 13
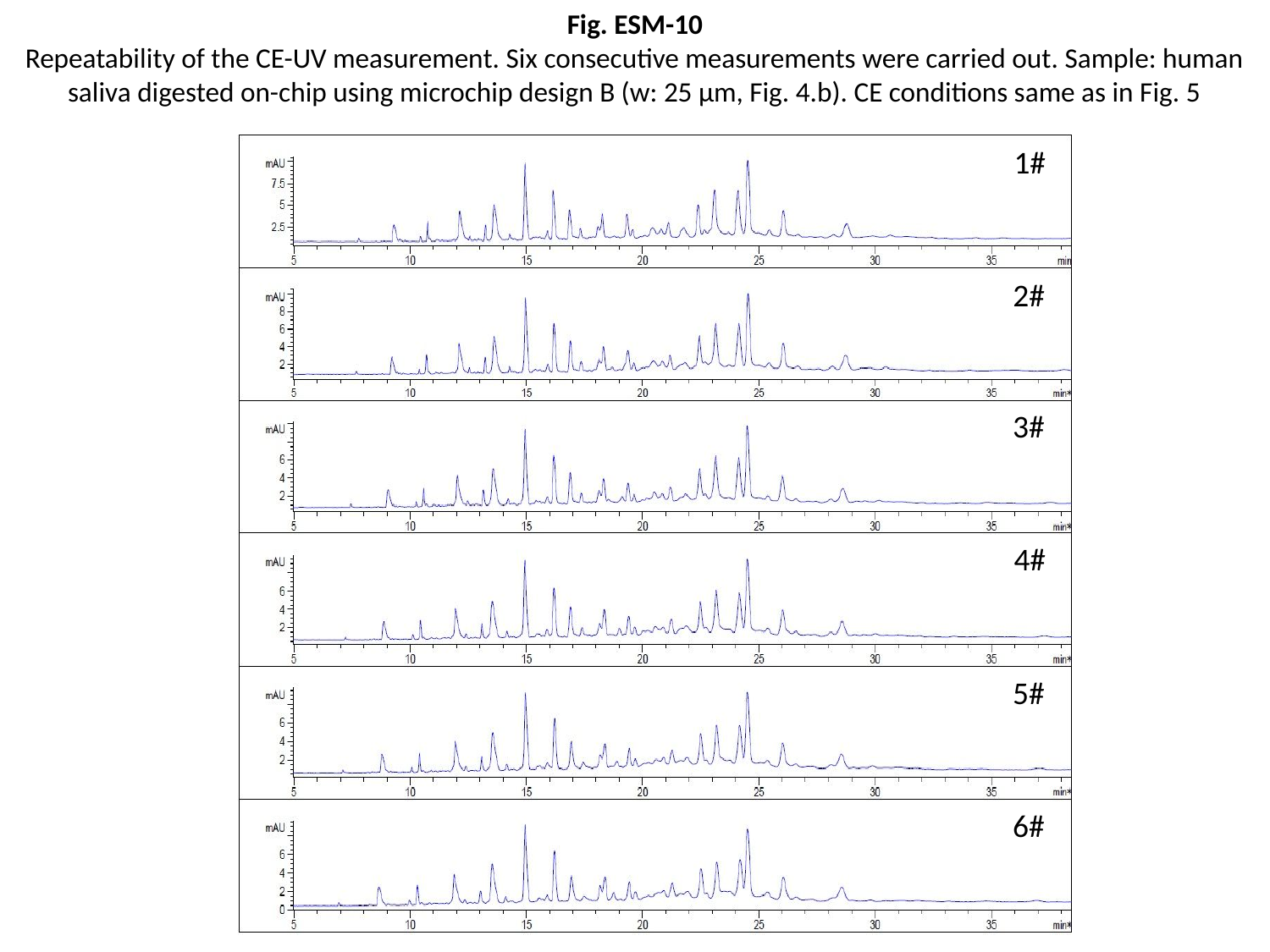

Fig. ESM-10
Repeatability of the CE-UV measurement. Six consecutive measurements were carried out. Sample: human saliva digested on-chip using microchip design B (w: 25 µm, Fig. 4.b). CE conditions same as in Fig. 5
1#
2#
3#
4#
5#
6#

## Slide 14
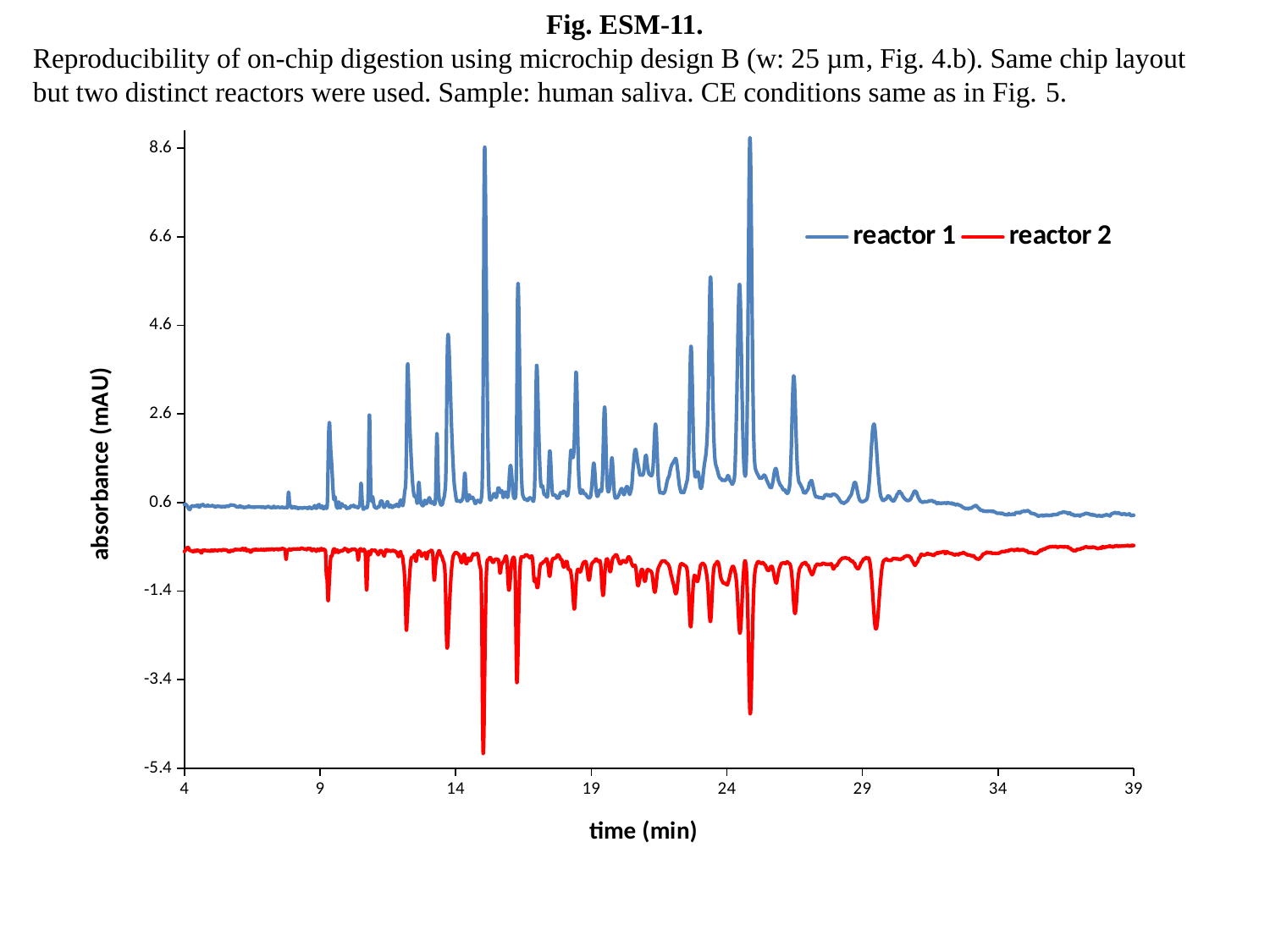

Fig. ESM-11.
Reproducibility of on-chip digestion using microchip design B (w: 25 µm, Fig. 4.b). Same chip layout but two distinct reactors were used. Sample: human saliva. CE conditions same as in Fig. 5.
### Chart
| Category | | |
|---|---|---|
